# Application of the *N*-Dibenzyl Protective Group in the Preparation of β-Lactam Pseudopeptides

**DOI:** 10.3390/molecules24071261

**Published:** 2019-04-01

**Authors:** Rok Frlan, Martina Hrast, Stanislav Gobec

**Affiliations:** The Chair of Pharmaceutical Chemistry, Faculty of Pharmacy, University of Ljubljana, Aškerčeva 7, 1000 Ljubljana, Slovenia; rok.frlan@ffa.uni-lj.si (R.F.); martina.hrast@ffa.uni-lj.si (M.H.)

**Keywords:** β-lactams, dibenzylamino, Mitsunobu reaction, nocardicin, pseudopeptides

## Abstract

Despite the great importance of β-lactam antibiotics, there is still a limited number of synthetic approaches for the formation of β-lactam–containing dipeptides. In this study, we report upon the stereoselective preparation of β-lactam–containing pseudopeptides, where different reaction conditions and NH_2_ protective groups were tested to obtain compounds that contain 3-amino-azetidin-2-one. We demonstrate that the protective group is essential for the outcome of the reaction. Successful implementation of dibenzyl-protected serine-containing dipeptides through the Mitsunobu reaction can provide the desired products at high yields and stereoselectivity.

## 1. Introduction

The β-lactam heterocycle is the main building block of the most broadly used antibacterial agents such as the penams (penicillin), penems, carbapenems, cephems and monobactams. Moreover, monocyclic β-lactams are potent cholesterol absorption inhibitors, which act through the inhibition of the enzyme cholesterol acyl transferase [1]. They are also dopamine receptor antagonists [2] and vasopressin V1a antagonists [3]. Spirocyclic β-lactams have also been shown to be potent type II β-turn–inducing peptide mimetics [4]. In addition, these heterocycles also have several other biological activities, such as antitumor, antitubercular, hypoglycemic, and anti-inflammatory actions. Their pharmacological and chemical activities greatly depend on the types of substitution at the β-lactam ring. However, synthesis of compounds with an appropriately substituted β-lactam ring with the desired diastereoselectivity is a challenging task for medicinal chemists [5,6].

There are numerous approaches for the synthesis of the β-lactam ring. The most frequently applied synthetic strategies include cycloaddition reactions, such as the Staudinger or Gilman-Speeter reactions, the Kinugasa reaction, or different cyclization reactions [7,8]. These last reactions can occur through formation of N1-C2, N1-C4, or C3-C4 bonds. Interestingly, the Mitsunobu reaction, which is the subject of the present report, has rarely been applied for the formation of the N1-C4 bond [9,10].

**Scheme 1:**
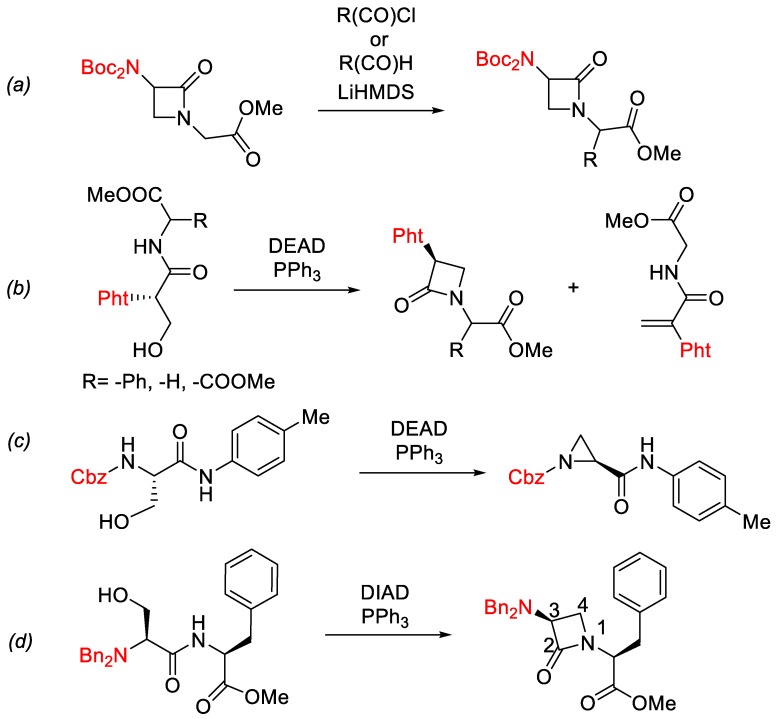
Formation of β-lactam pseudopeptides without a C4 substituent. Previous work: (**a**) alkylation of CH_2_, (**b**) phthalimide, Ox protective group, (**c**) Cbz, Boc protective group and (**d**) this work. DEAD = diethyl azodicarboxylate; DIAD = diisopropyl azodicarboxylate.

In this study, we focused on N1-C4 β-lactam ring closure with less common reactants: dipeptides. There are only a few methodologies in the literature that describe the formation of β-lactam pseudopeptides without a substituent on the β-lactam ring [10,11,12,13,14,15,16,17,18,19]. Versatile and efficient synthetic routes for β-lactam rings that contain dipeptides are thus needed. Recently, a new pathway for the synthesis of these compounds was developed that involves N-alkylation of the β-lactam ring with a haloacetate electrophile followed by the introduction of a side chain in the α-position with respect to the ester carbonyl group (Scheme 1a) [15]. However, despite a number of different compounds being synthesized by this methodology, it was not possible to form the desired monoalkylated products stereoselectively. In addition, there were also limitations in the selection of the electrophiles used for alkylation, which affected the scope of the reaction [15]. A more versatile procedure is therefore required to widen the chemical space of the structurally novel 3-amino-azetidin-2-ones.

**Figure 1:**
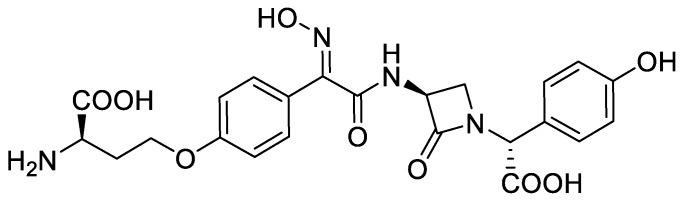
Nocardicin A, a representative β-lactam antibiotic of the nocardicins subgroup.

Previous reports of the preparation of nocardicins (Figure 1) have indicated *N*-phthalimido–protected serylphenylglycine dipeptides cyclized into azetidin-2-ones under the classical Mitsunobu reaction conditions (Scheme 1b). However, the dehydropeptide product and epimerization at the exocyclic chiral center (Cα’-H) were commonly observed [10]. Interestingly, the cyclization reaction was demonstrated to be strongly dependent on the acidity of the Cα’-H bond, rather than on the acidity of the N-H bond. This was rationalized by the hypothesis that a cyclization intermediate is formed in which the α’-C is sp2-hybridized and this increases the acidity of the nitrogen center. In general, increased acidity of Cα’-H facilitates the formation of β-lactams, and lowers the amount of dehydropeptides in the reaction mixture [16,17]. Simple serylphenylglycine dipeptides thus gave much lower yields compared to those obtained by cyclization of their phenylglycine analogs [17]. Direct application of synthetic methodologies developed for the synthesis of nocardicins and their analogs is therefore not directly applicable to the preparation of β-lactam phenylalanine derivatives. 

For the successful reaction here, protection of both of the hydrogens of the primary amine functionality in the serine was demonstrated to be of vital importance. When the carboxybenzyl (Cbz) or *tert*-butyloxycarbonyl (Boc) protective groups were applied, the reaction resulted in only a mixture of aziridine and dehydropeptide (Scheme 1c, Cbz, Boc, respectively). The use of phthalimide (Pht) or 4,5-diphenyloxazoline-2-one (Ox) protective groups is thus essential for the successful reaction (Scheme 1b) [16,17,20].

## 2. Results and Discussion

During an ongoing project that is devoted to the preparation of pharmacologically interesting monocyclic β-lactam analogs (e.g., Figure 1, the nocardicins), we initially faced the task of synthesizing β-lactam phenylalanine derivatives that differ from nocardicins in their additional CH_2_ group between the phenyl ring and the exocyclic Cα’-H group. 

**Scheme 2:**
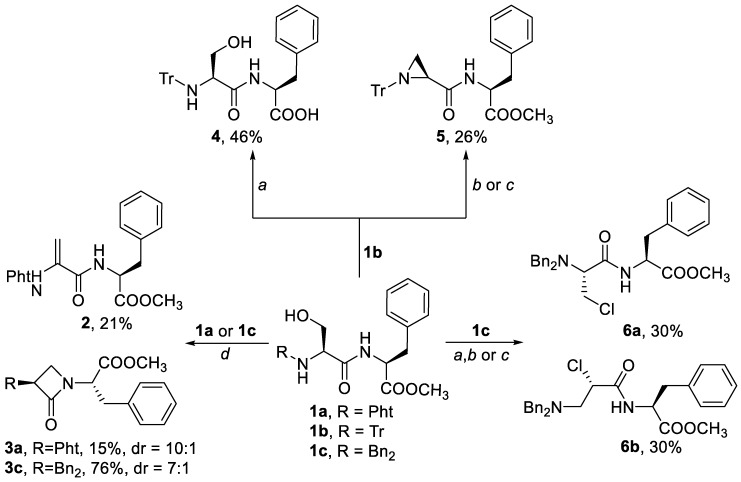
N1-C4 cyclization of dipeptide **1a–1c**. Conditions: (a) KOH, *p*TolSO_2_Cl, CH_2_Cl_2_, reflux; (b) SO_2_Cl_2_, Et_3_N, toluene, −50 °C to rt; (c) CH_3_SO_2_Cl, Et_3_N, DMAP, CH_2_Cl_2_; (d) DIAD, PPh_3_, THF, 0 °C to rt, 16 h, 15% yield or DIAD, P(OEt)_3_, THF, rt, 16 h, 0% yield.

The initial aim of this study was to find the optimal reaction conditions to prepare β-lactam pseudopeptides by the N1-C4 cyclization of serylphenylalanine derivatives, such as **1a** (Scheme 2). The effects of various *N*-protecting groups on the reaction outcome, and the application of these findings to other chemically similar dipeptides under different reaction conditions, were first screened. A hypothesis was defined such that because of the steric and electronic properties of a protective group, it would be essential to the formation of both the β-lactam ring and the amounts of the dehydrated products. 

Three very different protective groups were applied on the primary serine NH_2_ group, each with very distinctive electron-withdrawing properties that could potentially influence the reaction outcome: phthalimide (**1a**), trityl (**1b**) and dibenzyl (**1c**) protective groups. Both, phthalimide (**1a**) and dibenzyl (**1c**) protective groups were used to mask hydrogens of the primary NH_2_ in order to prevent the well-documented risk of aziridine formation in the cyclodehydration step [20]. In contrast, the application of the trityl protection was rationalized by the idea that its unmasked N-H group would not participate in the following reactions, because of its low acidity and the steric hindrance of the triphenylmethane moiety. In addition, both trityl (**1b**) and dibenzyl (**1c**) protective group also have lower electron withdrawing properties compared to the phthalimide group (**1a**). 

Next, each of the three compounds **1a**–**1c** was treated under different reaction conditions. The idea was also to avoid high reaction temperatures and strong bases, such as lithium diisopropylamide (LDA) or NaH, which have otherwise been used in other β-lactam ring preparations [11,21]. Firstly, the conversion of an alcoholic OH group of serine to a sulfonate group was tested because it is widely used in aziridine ring formation, in which carbamate or trityl nitrogen is used as the nitrogen source [22,23]. Furthermore, the activation of a serine OH group via conversion to an imidazolyl sulfonate has been reported to yield 3-aminonocardicinic acids [24,25]. It was therefore postulated that each of the derivatives **1a–1c** will react with the amide nitrogen instead. For this purpose, three reagents were used: *p*-toluenesulfonyl chloride (condition a); sulfuryl chloride (condition b); and methanesulfonyl chloride (condition c). Unfortunately, all three reactions by which a sulfonate was formed (i.e., conditions a, b, c) as an intermediate were not suitable for β-lactam ring formation. These conditions yielded only a product of ester hydrolysis **4**, the aziridine **5** or a mixture of chlorides **6a** and **6b** in a ratio of 1:1 (which we were not able to separate) (Scheme 2).

Secondly, compound **1a** was treated under Mitsunobu reaction conditions, which is commonly applied for the preparation of nocardicin derivatives. However, to the best of our knowledge, there are no previous reports on the preparation of β-lactam phenylalanine derivatives using the Mitsunobu reaction. Furthermore, attempts to prepare less reactive glycine derivatives using the Mitsunobu reaction have given the corresponding β-lactam ring at poor yields. In addition, the mixtures obtained were difficult to separate, which was explained by the lower acidity of the exocyclic Cα’-H group which consequently decreases the yield of a reaction and increases the number of side products [16]. It was therefore not surprising that our first attempts to cyclize **1a** (Scheme 2) under the classical Mitsunobu conditions using PPh_3_/DIAD or DEAD gave only low yields of β-lactam **3a** and dehydropeptide **2**, in nearly equimolar ratios. These are difficult to separate in larger quantities. In addition, the application of P(OEt)_3_ instead of PPh_3_ in the Mitsunobu reaction, which has been successfully applied in the synthesis of nocardicin analogs, resulted in no products whatsoever [13,18]. Our findings were therefore in agreement with the literature reports that have described the influence of the Cα’-H group acidity on the reaction outcome (Scheme 1) [16]. 

Further optimization of the reaction conditions through changing the solvent and temperature increased the yield of β-lactam **3a** to 45%, but the ratio of dehydrodipeptide **2** to **3a** remained at 1:1. A modified procedure that would give larger quantities of β-lactam using simple cyclization without the formation of dehydrodipeptide was therefore required. Compounds **1b** and **1c** with trityl and dibenzyl protection were then treated under the same classical Mitsunobu reaction conditions as **1a**. Even though the tritylated compound **1b** gave no product whatsoever, a combination of the classical Mitsunobu reaction and dibenzyl protection, gave positive results, whereby dipeptide **1c** was transformed into the desired *SS* diastereoisomer **3c** at 76% isolated yield. Epimerization at the exocyclic Cα’-H stereogenic center was also seen, with the diastereoisomeric ratio (*dr*) of *SS* to *SR* of 7:1; these were easy to separate. Importantly, no dehydrodipeptide side products were seen, and the yields were much higher when compared to the results of the Mitsunobu reaction for phthalimide protected dipeptide **1a**. Furthermore, the outcome of the reaction gave higher isolated yields when compared to the less reactive phthalimide-protected glycine [16] and cyclopropyl derivatives [17] that have been reported in the literature.

**Scheme 3:**
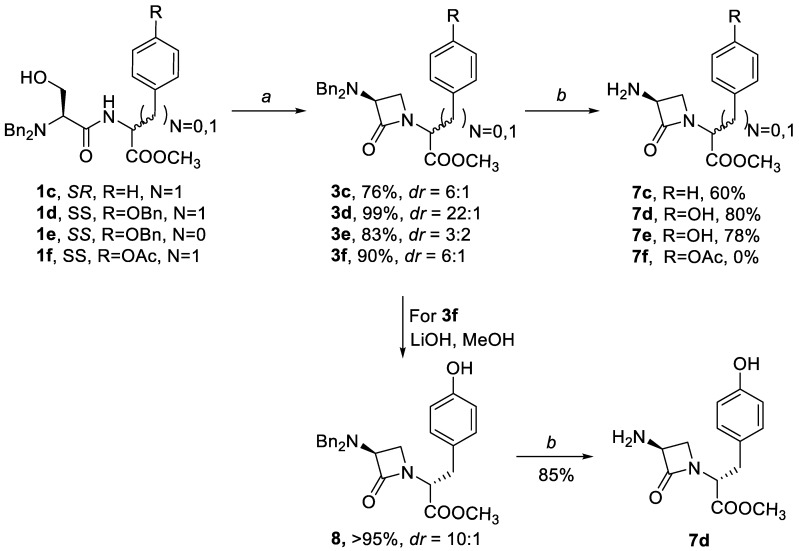
Formation of β-lactam pseudopeptides and their deprotection. Conditions: (a) DIAD, PPh_3_, THF or DMF; (b) H_2_-Pd/C, 5% EtOH/EtOAc.

The reaction conditions for both of the reaction steps were then further tested on analogous serylphenylalanine dipeptides **1d, 1e**, which both gave cyclized products **3d**, **3e** at 99% and 90% yields, respectively (Scheme 3). These demonstrated the utility of the dibenzyl protective group in the preparation of simple β-lactam phenylalanine derivatives. 

However, there are also limitations when the acidity of the Cα stereogenic center is higher, in terms of the *dr* and the optimal solvent. For example, phenylalanine **3c**, benzyl **3d**, and acetyl-protected tyrosine dipeptide **3f** were all prepared at high *dr* in THF. In contrast, cyclization of the phenylglycine derivative **1e** in which a direct link between Cα’-H and electron-withdrawing phenyl ring makes Cα’-H more acidic compared to the phenylalanine analogues **1c**, **1d** and **1f**, proceeded only in DMF, to give **3e** at high yields, but with almost equimolar *dr* of the *SS* and *SR* isomer. It appears that the basic nitrogen of the dibenzylamino moiety in combination with DMF as a solvent induces epimerization at the exocyclic Cα’-H when the basic dibenzylamino moiety is applied. This is in agreement with a report by Miller, who observed epimerization to thermodynamic equilibrium when traces of trimethylamine were added to pure phenylglycine derivatives [13]. 

Removal of the dibenzyl protecting group from **3b**–**3e** preceded the next reaction step. Pd-C in a mixture of 5% EtOH in ethyl acetate was preferred to other combinations of catalysts and solvents because it gave significantly higher yields compared to the reactions in EtOH, MeOH or THF. Here, the percentage of EtOH and the traces of DIAD in the reaction mixtures had an enormous influence on the yield and the general outcome of the reactions. In the presence of DIAD as an impurity, higher amounts of EtOH had to be used to obtain full conversion of **3b**–**3f** to yield the corresponding products **7b**–**7f**. However, higher proportions of EtOH also resulted in lower yields, with higher amounts of side products. The purities of all of the compounds **3b**–**3f** were thus of uttermost importance for the successful reaction (Scheme 3).

Compounds **3c**–**3e** were deprotected with 100% conversion to yield debenzylated products **7b**, **7d**, and **7e** at 60%, 80%, and 78% isolated yields, respectively (Scheme 3). Many other side products were also observed, among which l-Phe-OCH_3_ was isolated at 10% yield when **3b** was treated under these conditions. Unfortunately, the acetyl protective group on phenol **3f** proved to be too labile to enable successful deprotection to **7f**, giving instead only a mixture of unidentifiable acetylated products. However, it was easy to selectively remove acetyl protection from the tyrosine OH group prior to debenzylation of **3e**. For this purpose, 0.1 M LiOH in methanol was used, to obtain phenol **8** at quantitative yields, with *dr* of 10:1. This product offered the possibility of future preparation of *O*-derivatized analogs. This compound was then easily debenzylated to **7d** at 85% yield. 

Because these compounds contain a primary amine and an ester functional group they have a potential to oligomerize after prolonged storage. We would therefore advise the reader to transform them into some form of a salt if stored for a prolonged period of time.

## 3. Materials and Methods

### 3.1. Materials and Instruments

The reactions were monitored by TLC carried out on Merck silica gel (60 F254, Darmstadt, Germany) by using UV light as visualizing agent, KMnO_4_ in water, phosphomolybdic acid in ethanol and ninhydrine in ethanol and heat as developing agents. Column chromatography was performed using Merck Silica Gel 60. Proton nuclear magnetic resonance spectra (^1^H-NMR) were obtained at 400 MHz on Bruker 400 spectrometers (Billerica, MA, USA). Spectra were recorded in CDCl_3_, MeOD and DMSO-*d*_6_ solutions. Chemical shifts are reported in ppm, referenced to tetramethylsilane (TMS) as the external reference. Carbon-13 nuclear magnetic resonance spectra (^13^C-NMR) were obtained at 100 MHz on Bruker 400 spectrometer. Chemical shifts are reported in ppm, referenced to the solvent peak of CDCl_3_. Low-resolution mass spectra were obtained with a Shimadzu GC-MS-QP2010 mass spectrometer (Kyoto, Japan). High resolution mass spectra (HRMS) were recorded on Q Executive Plus LC-MS/MS system (Thermo Scientific, Waltham, MA, USA).

### 3.2. Synthesis and Characterization

#### 3.2.1. Procedure for Synthesis of Precursors **1a** and **1b** (Scheme 2)

*(S)-Methyl ((S)-2-((tert-butoxycarbonyl)amino)-3-hydroxypropanamido)-3-phenylpropanoate* (**S3**) [26]. To a suspension of Boc-L-Ser (**S1**) (1.00 g, 4.9 mmol, 1.0 eq) in CH_2_Cl_2_ (50 mL, 0.1 M), *N*-methylmorpholine was added (1.6 mL, 14.6 mmol, 3.0 eq), followed by the addition of l-Phe-OCH_3_ hydrochloride (**S2**) (1.05 g, 4.9 mmol, 1.0 eq). After 0.5 h, the solution was cooled to 0 °C and HOBt (658 mg, 4.9 mmol, 1.0 eq) and EDC (1.02 g, 5.4 mmol, 1 eq) were added and the reaction mixture was stirred for 18 h at room temperature. A solution was dissolved in ethyl acetate (100 mL) and extracted with 10% citric acid (2 × 20 mL), saturated solution NaHCO_3_ (2 × 20 mL), brine (20 mL) and dried over anhydrous Na_2_SO_4_. Organic solvent was evaporated under reduced pressure and the crude product was purified by flash column chromatography in Hex/EtOAc = 1/2 as eluent. White solid (446 mg, 1.2 mmol, 25% yield) [26]; R_f_ (Hexane/ethyl acetate = 1/2) = 0.25; mp = 92–93 °C; ^1^H-NMR (400 MHz, DMSO-*d*_6_): *δ* 8.14 (d, *J* = 7.8 Hz, 1H), 7.31–7.14 (m, 5H), 6.65 (d, *J* = 8.3 Hz, 1H), 4.80 (t, *J* = 5.8 Hz, 1H), 4.49 (ddd, *J* = 8.0, 7.8, 5.8 Hz, 1H), 3.99 (ddd, *J* = 8.3, 6.8, 4.7 Hz, 1H), 3.58 (s, 3H), 3.50, 3.43 (ABXY, *J* = 11.2, 5.8, 4.7, 6.8 Hz, 2H), 3.01, 2.94 (ABX, dd, *J* =13.5, 8.0, 5.8 Hz, 2H) ppm; ^13^C-NMR (100 MHz, CDCl_3_): *δ* 172.0, 171.2, 135.9, 129.4, 128.8, 127.4, 80.6, 63.1, 55.2, 53.6, 52.7, 37.9, 28.4 ppm; MS (ESI^+^): *m*/*z* 389 [M + Na]^+^, 289 (100); HRMS (ESI^−^): Calc mass for C_18_H_25_N_2_O_6_ [M − H]^−^: 365.1713; measured: 365.1721; IR (ATR): 3521.6, 3338.0, 2977.8, 1736.1, 1668.1, 1649.2, 1540.3, 1519.9, 1307.3, 1266.4, 1223.9, 1008.3, 698.4, 616.3 cm^-1^; ${\left[\alpha \right]}_{D}^{25}$ = +33.7 (0.56, MeOH).

*(S)-Methyl 2-((S)-2-amino-3-hydroxypropanamido)-3-phenylpropanoate hydrochloride* (**S4**) [26]. Compound **S4** was synthesized according to the literature procedure [26]. To a solution of **S3** (446 mg, 1.2 mmol) in CH_2_Cl_2_ (5 mL) was added CF_3_COOH (1 mL) dropwise at 0 °C, and the solution was stirred at rt for 5 h. Solvent was evaporated and the white residue was dissolved in diethyl ether (10 mL) to which 2 M HCl in diethyl ether (3.0 mL, 6.1 mmol, 5 eq) was added and filtered to afford the product **S4**. White needles (306 mg, 1.0 mmol, 83% yield); mp = 149–150 °C; ^1^H-NMR (400 MHz, MeOD): *δ* 7.36–7.19 (m, 5H), 4.75 (dd, *J* = 8.7, 5.5 Hz, 1H), 4.04–3.87 (m, 2H), 3.79 (dd, *J* = 10.3, 5.9 Hz, 1H), 3.73 (s, 3H), 3.23 (dd, *J* = 14.2, 5.4 Hz, 1H), 3.04 (dd, *J* = 14.2, 8.7 Hz, 1H) ppm; ^13^C-NMR (100 MHz, MeOD): *δ* 173.1, 168.4, 138.1, 130.4, 129.7, 128.2, 61.8, 56.3, 55.7, 53.0, 38.3 ppm; MS (ESI^+^): *m*/*z* 289 ([M + Na]^+^, 100), 267 ([M + H]^+^, 80); IR (ATR): 3325.0, 3245.5, 1731.4, 1663.4, 1535.3, 1445.8, 1363.2, 1225.6, 1049.1, 699.2, 617.6 cm^−1^; ${\left[\alpha \right]}_{D}^{25}$ = +86.0 (0.233, MeOH).

*(S)-Methyl ((S)-2-(3-(1,3-dioxoisoindolin-2-yl)-2-oxoazetidin-1-yl)-3-phenylpropanoate* (**1a**, Scheme 2). To a solution of **S4** (1.1 g, 3.63 mmol, 1 eq) in DMF (20 mL) phthalanhydride (0.59 g, 3.99 mmol, 1.1 eq) and triethylamine (0.53 mL, 3.81 mmol, 1.05 eq) were added, and the solution was stirred at 100 °C for 18 h. After the mixture was cooled to room temperature, DMF was evaporated under reduced pressure and the oily residue was dissolved in EtOAc (50 mL). Organic phase was washed with water (50 mL), 1 M HCl (50 mL), saturated solution NaHCO_3_ (50 mL) and brine (50 mL) and dried over anhydrous Na_2_SO_4_. Organic solvent was evaporated under reduced pressure yielding a white solid. White solid (1.14 g, 2.9 mmol, 80% yield); R_f_ (hexane/ethyl acetate = 1/1) = 0.11; mp = 93–96 °C; ^1^H-NMR (400 MHz, CDCl_3_): δ 7.85 (dd, *J* = 5.5, 3.1 Hz, 2H), 7.73 (dd, *J* = 5.5, 3.1 Hz, 2H), 7.33–7.37 (m, 2H), 7.27–7.30 (m, 1H), 7.22–7.24 (m, 2H), 5.25 (dd, *J* = 5.6, 3.0 Hz, 1H,), 4.87 (dd, *J* = 9.3, 6.2 Hz, 1H), 3.90 (dd, *J* = 5.4, 3.0 Hz, 1H), 3.83 (s, 3H), 3.64 (t, *J* = 5.6 Hz, 1H), 3.29 (dd, *J* = 14.3, 6.2 Hz, 1H), 3.08 (dd, *J* = 14.3, 9.3 Hz, 1H) ppm; ^13^C-NMR (100 MHz, CDCl_3_): 135.8, 134.4, 131.7, 128.9, 128.7, 127.7, 123.3, 55.0, 53.1, 52.6, 45.2, 35.8 ppm; MS (ESI^+^): *m*/*z* 419 [M + Na, 100]^+^; HRMS (ESI^+^): Calc mass for C_21_H_21_O_6_N_2_: 397.1394 [M + H]^+^; measured: 397.1406; IR (ATR): 1711.4, 1675.8, 1529.2, 1467.8, 1437.5, 1386.1, 1215.2, 1115.5, 1079.7, 1030.3, 983.2, 911.3, 877.3, 789.1, 719.7, 702.4, 648.9, 531.4, 504.7 cm^−1^; ${\left[\alpha \right]}_{D}^{25}$ = −213.0 (0.424, MeOH).

*Methyl trityl-l-seryl-l-phenylalaninate* (**1b,** Scheme 2). To a solution of dipeptide **S4** (500 mg, 1.7 mmol, 1.0 eq) in chloroform (5.5 mL, 0.3 M), trimethylamine (690 μL, 5.0 mmol, 3 eq) was added. After 5 min, the solution was cooled to 0 °C followed by the slow addition of trityl chloride (576 mg, 2.0 mmol, 1.2 eq) at 0 °C. The reaction mixture was stirred for 18 h at room temperature. Ethyl acetate (50 mL) was added and the mixture was extracted with 10% citric acid (2 × 10 mL), saturated solution NaHCO_3_ (2 × 10 mL), brine (10 mL) and dried over anhydrous Na_2_SO_4_. Organic solvent was evaporated under reduced pressure and the crude product was recrystallized from diethyl ether. White crystals (220 mg, 432 mmol, 26% yield); R_f_ (hexane/ethyl acetate = 1/1) = 0.29; mp = 118–120 °C; ^1^H-NMR (400 MHz, CDCl_3_): δ 7.79 (d, *J* = 8.5 Hz, 1H), 7.47–7.12 (m, 20H), 4.85 (ddd, *J* = 8.5, 6.3, 6.0 Hz, 1H), 3.79 (s, 3H), 3.48, 2.32 (AMXY, *J* = 11.1, 5.5, 2.8, 5.7 Hz, 2H), 3.21 (bs, 1H), 3.16, 3.13 (ABX, *J* = 14.6, 6.0, 6.3 Hz, 2H), 2.80 (bs, 1H), 1.67 (t, *J* = 5.5 Hz, 1H) ppm; ^13^C-NMR (100 MHz, CDCl_3_): *δ* 173.7, 172.2, 146.0, 136.1, 129.5, 129.0, 128.8, 128.3, 127.5, 126.9, 71.7, 63.4, 59.1, 53.0, 52.8, 38.1 ppm; MS (ESI^+^): *m*/*z* 531 ([M + Na]^+^, 100); HRMS (ESI^−^): Calc mass for C_32_H_31_N_2_O_4_ ([M − H]^−^, 100): 507.2284; measured: 507.2277; IR (ATR): 3593.4, 3468.1, 332.0, 2948.7, 1739.8, 1668.0, 1491.1, 1198.9, 1016.0, 743.6, 706.1 cm^−1^; ${\left[\alpha \right]}_{D}^{25}$ = +198.4 (0.310, MeOH).

#### 3.2.2. Procedure for Synthesis of Precursor **1c** (Scheme 2)

*Dibenzyl-l-serine***(S6)** [27]. Dibenzyl-l-serine (**S6**) was synthesized according to the modified literature procedure [27]. l-Ser (20.0 g, 190 mmol, 1 eq) and tetrabutylammonium iodide (7.0 g, 19 mmol, 0.1 eq) were dissolved in a solution of KOH (74.7 g, 1.3 mol, 7 eq, 3 M) in a mixture of water and ethanol (1:1, 440 mL). Benzyl chloride (131 mL, 1.14 mmol, 6 eq) was added dropwise and the mixture was stirred under reflux for 1 h. KOH (10.7 g, 190 mmol, 1 eq) was added again and the mixture was stirred further for another hour. After the mixture was cooled down, the white precipitate was dissolved by the addition of water. The solution was extracted with toluene (5 × 100 mL), combined organic fractions were washed with brine (50 mL) and dried over anhydrous Na_2_SO_4_. Organic solvent was evaporated under reduced pressure and the crude product was purified by flash column chromatography. The crude product was dissolved in water (150 mL), the pH value was adjusted to 5 with 1 M HCl and the mixture was left standing over 2 days in a refrigerator to obtain white crystals. White crystals (41.2 g, 144 mmol, 76% yield); mp = 144–146 °C; ^1^H-NMR (400 MHz, DMSO-*d*_6_): δ 7.40–7.14 (m, 10H), 3.81, 3.62 (AB, *J* = 13.9 Hz, 2H), 3.78, 3.64 (ABX, *J* = 11.0, 7.2, 6.0 Hz, 2H), 3.25 (dd, *J* = 7.2, 6.0 Hz, 1H) ppm.

*Methyl dibenzyl-l-seryl-l-phenylalaninate* (**1c**, Scheme 2). Dipeptide methyl dibenzyl-l-seryl-l-phenylalaninate (**1b**) was synthesized in a coupling procedure similar to the synthesis of **S3** starting from dibenzyl-l-serine (**S6**) (1.10 g, 3.9 mmol, 1.0 eq). The crude product was purified by flash column chromatography on silica using a gradient of hexane/ethyl acetate starting from 3/1 to 1/1. Colorless liquid (1.62 g, 3.6 mmol, 94% yield); R_f_ (Hexane/ethyl acetate = 3/1) = 0.18; ^1^H-NMR (400 MHz, CDCl_3_): δ 7.94 (d, *J* = 7.7 Hz, 1H), 7.33–7.13 (m, 10H), 7.13–6.95 (m, 5H), 4.83 (ddd, *J* = 7.7, 5.3, 6.2 Hz, 1H), 4.03, 3.95 (ABX, *J* = 11.3, 6.9, 4.4 Hz, 2H), 3.78 (s, 3H), 3.73, 3.49 (AB, *J* = 13.7 Hz, 4H), 3.31 (dd, *J* = 6.9, 4.4 Hz, 1H), 3.26, 3.08 (AB, *J* = 14.0, 5.3, 6.2 Hz, 2H) ppm; ^13^C-NMR (100 MHz, CDCl_3_): *δ* 173.6, 171.9, 138.4, 135.7, 129.2, 128.7 (2 signals overlapping), 128.6, 127.5, 127.2, 62.4, 58.1, 54.9, 53.3, 52.6, 38.1 ppm; MS (ESI^+^): *m*/*z* 469 [M + Na]^+^; HRMS (ESI^−^): Calc. Mass for C_27_H_29_N_2_O_4_: 445.2127 ([M − H]^−^, 100), measured: 445.2130; IR (ATR): 3372.9, 3028.5, 1742.6, 1655.4, 1494.6, 1216.0, 1028.0, 697.8 cm^−1^; ${\left[\alpha \right]}_{D}^{25}$ = +133.6 (0.44, MeOH).

#### 3.2.3. Synthesis of Trityl Protected Dipeptide **4** and Aziridine **5** (Scheme 2)

*Trityl-l-seryl-l-phenylalanine* (**4**, Scheme 2). To a solution of trityl protected dipeptide **1b** (0.1 g, 0.2 mmol, 1 eq) in CH_2_Cl_2_ (10 mL), TsCl (1.1 eq) and KOH (2.5 eq) were added and the reaction mixture was stirred for 4 h at 50 °C. The solution was cooled to room temperature, organic solvent was evaporated and the crude mixture was purified by flash column chromatography on silica using ethyl acetate/methanol = 5/1 as eluent. White solid (45 mg, 0.09 mmol, 46% yield); R_f_ (Hexane/ethyl acetate = 1/1) = 0; mp = 71–37 °C; ^1^H-NMR (400 MHz, CDCl_3_): δ 7.47–7.12 (m, 21H), 4.62–4.42 (m, 1H), 3.52, 2.50 (m, 2H), 3.23–3.13 (m, 1H), 3.12–2.94 (m, 2H) ppm; ^13^C-NMR (100 MHz, CDCl_3_): *δ* 175.8, 174.8, 145.9, 136.3, 129.5, 129.0,128.8, 128.2, 127.4, 126.9, 71.6, 63.6, 58.9, 54.0, 37.3 ppm; MS (ESI^−^): *m*/*z* 493 [M − H, 100]^+^; HRMS (ESI^−^): Calc mass for C_31_H_29_N_2_O_4_ [M − H]^−^: 493.2133; measured: 493.2135; IR (ATR): 3327.3, 2928.2, 1723.9, 1649.4, 1520.1, 1492.8, 1447.5, 1187.6, 1123.2, 1030.1, 903.6, 745.7, 698.3, 633.4, 523.1 cm^−1^.

*Methyl ((S)-1-tritylaziridine-2-carbonyl)-l-phenylalaninate* (**5**, Scheme 2) [28]. Procedure A: To a solution of dipeptide (0.1 g, 0.2 mmol, 1 eq) in anhydrous toluene, triethylamine (68 µL, 0.5 mmol, 2.5 eq) was added under argon atmosphere. The reaction mixture was cooled to −50 °C and SO_2_Cl_2_ was added dropwise. The mixture was stirred at −50 °C for 2 h, then 18 h at room temperature. The solvent was evaporated and the crude product was purified by flash chromatography. Procedure B: To a solution of dipeptide (0.1 g, 0.2 mmol, 1 eq) in CH_2_Cl_2_ (10 mL) cooled to 0 °C, triethylamine (68 µL, 0.49 mmol, 2.5 eq) was added, followed by MsCl (17 µL, 0.21 mmol, 1.1 eq). The reaction mixture was stirred at room temperature for 16 h. The solvent was evaporated and the crude product was purified by flash chromatography using a gradient of hexane/ethyl acetate starting from 4/1 to 1/1. White solid (25 mg, 0.05 mmol, 26% yield); R_f_ (Hexane/ethyl acetate = 1/1) = 0.35; mp = 53–55 °C; ^1^H-NMR (400 MHz, CDCl_3_): δ 7.34–7.37 (m, 7H), 7.16–7.29 (m, 13H), 4.90–4.96 (m, 1H), 3.80 (s, 3H), 3.20 (*J* = 6.1, 1.8 Hz, 2H), 1.95 (dd, *J* = 6.6, 2.7 Hz, 1H), 1.69 (dd, *J* = 2.6, 0.7 Hz, 1H), 1.37 (dd, *J* = 6.6, 0.7 Hz, 1H) ppm; ^13^C-NMR (100 MHz, CDCl_3_): *δ* 171.0, 170.6, 143.4, 136.0, 129.5 (two overlapping signals), 128.9, 127.9, 127.5, 127.3, 74.7, 52.7, 52.1, 38.1, 34.0, 30.1 ppm; MS (ESI^+^): *m*/*z* 513 [M + Na, 100]^+^; MS (ESI^−^): *m*/*z* 489 [M − H, 100]^−^; HRMS (ESI^+^): Calc mass for C_32_H_31_O_3_N_2_ [M + H]^+^: 491.2329; measured: 491.2332; IR (ATR): 3367.6, 3058.4, 1743.5, 1677.2, 1511.4, 1446.8, 1351.4, 1216.6, 1080.7, 1009.2, 902.8, 745.1, 705.9, 633.4 cm^−1^; ${\left[\alpha \right]}_{D}^{25}$ = −332.3 (0.546, MeOH).

#### 3.2.4. General Procedure for the Synthesis of **2**, **3a**, **3c** via Mitsunobu Reaction (Scheme 2 and Scheme 3)

To a solution of dipeptide **1a** or **1c** (0.2 mmol, 1.0 eq) and PPh_3_ (1.5 eq) in anhydrous tetrahydrofuran DIAD (1.5 eq) was added drop-wise at 0 °C under argon atmosphere. After the solution was stirred for 18 h at room temperature the solvent was evaporated and the crude product was purified by flash chromatography.

*Methyl (2-(1,3-dioxoisoindolin-2-yl)acryloyl)-l-phenylalaninate* (**2**, Scheme 2). White solid (20 mg, 0.052 mmol, 21% yield; R_f_ (Hexane/ethyl acetate = 1/2) = 0.51; mp = 63–65 °C; ^1^H-NMR (400 MHz, CDCl_3_): δ 7.91 (dd, *J* = 5.5, 3.1 Hz, 2H), 7.78 (dd, *J* = 5.5, 3.1 Hz, 2H), 7.21–7.30 (m, 3H), 7.15–7.17 (m, 5H), 6.48 (d, *J* = 7.6 Hz, 1H), 6.08 (d, *J* = 1,4 Hz, 1H), 5.82 (d, *J* = 1,4 Hz, 1H), 4.96 (dt, *J* = 7.6, 5.3 Hz, 1H), 3.76 (s, 3H), 3.22 (t, *J* = 5.2 Hz, 2H) ppm; ^13^C-NMR (100 MHz, CDCl_3_): *δ* 171.6, 166.5, 162.6, 135.7, 134.7, 132.7, 131.8, 128.7, 128.6, 127.3, 124.1, 120.5, 53.7, 52.6, 37.7 ppm; MS (ESI^+^): *m*/*z* 401 [M + Na]^+^; MS (ESI^−^): *m*/*z* 377 [M − H]^+^; HRMS (ESI^+^): Calc mass for C_21_H_19_O_5_N_2_ [M + H]^+^ 379.1288; measured: 379.1287; IR (ATR): 3299.1, 1789.4, 1722.6, 1656.8, 1624.7, 1536.3, 1454.7, 1378.1, 1303.2, 1273.6, 1207.7, 1172.2, 1116.3, 1083.1, 1031.3, 995.4, 950.6, 920.1, 883.4, 788.1, 735.2, 701.3, 630.1, 572.4, 532.5 cm^−1^; ${\left[\alpha \right]}_{D}^{25}$ = −585,9 (0.375, MeOH).

*Methyl (S)-2-((S)-3-(1,3-dioxoisoindolin-2-yl)-2-oxoazetidin-1-yl)-3-phenylpropanoate* (**3a**, Scheme 2). White solid (14 mg, 0.037 mmol, 15% yield); R_f_ (chloroform/acetone = 100/5) = 0.39; mp =122–124 °C; ^1^H-NMR (400 MHz, CDCl_3_): *δ* 7.88 (dd, *J* = 5.5, 3.0 Hz, 2H), 7.77 (dd, *J* = 5.5, 3.0 Hz, 2H), 7.16–7.20 (m, 3H), 7.05–7.07 (m, 2H), 6.80 (d, *J* = 7.7 Hz, 1H), 4.86–4.91 (m, 2H), 4.40 (dd, *J* = 11.5, 6.8 Hz, 1H), 4.03 (dd, *J*= 11.5, 5.3 Hz, 1H), 3.73 (s, 3H), 3.13 (dd, *J* = 5.7, 4.1 Hz, 2H) ppm; ^13^C-NMR (100 MHz, CDCl_3_): δ 171.7, 168.0, 167.4, 135.6, 134.5, 131.7, 129.3, 128.6, 127.2, 123.7, 60.9, 54.5, 53.51, 52.5, 37.7 ppm; MS (ESI^−^): *m*/*z* 376.5 [M − H]^−^; MS (ESI^+^) *m*/*z* 400.6 [M + Na]^+^; HRMS (ESI^+^): Calc mass for C_21_H_19_O_5_N_2_ [M + H]^+^: 379.1288; measured: 379.1298; IR (ATR): 1763.4, 1740.4, 1717.2, 1391.3, 1205.2, 1115.8, 1025.9, 716.9, 701.6 cm^−1^; ${\left[\alpha \right]}_{D}^{25}$ = −507.8 (0.546, MeOH).

*Methyl (S)-2-((S)-3-(dibenzylamino)-2-oxoazetidin-1-yl)-3-phenylpropanoate* (**3c*_S_***, Scheme 2). Yellow oil (76% yield); R_f_ (hexane/ethyl acetate = 6/1) = 0.19; ^1^H-NMR (400 MHz, CDCl_3_): *δ* 7.39–7.11 (m, 16H), 4.70 (dd, *J* = 10.0, 5.6 Hz, 1H), 4.13 (dd, *J* = 5.2, 2.5 Hz, 1H), 3.74, 3.64 (AB, *J* = 13.7 Hz, 4H), 3.39, 3.18 (AMX, *J* = 5.6, 5.2, 2.5 Hz, 2H), 3.71 (s, 3H), 3.22, 3.00 (ABX, *J* = 13.4, 10.0, 5.6 Hz, 2H) ppm; ^13^C-NMR (100 MHz, CDCl_3_): δ 170.8, 169.7, 138.6, 136.4, 129.1, 128.9, 128.8, 128.5, 127.4, 127.3, 68.5, 54.8, 54.7, 52.7, 43.2, 35.7 ppm; MS (ESI^+^): *m*/*z* 429 [M + H]^+^; HRMS (ESI^−^): Calc mass for C_27_H_29_O_3_N_2_ [M − H]^−^: 429.2173; measured: 429.2170; IR (ATR): 3028.4, 2952.4, 1744.9, 1447.4, 1220.3, 744.2, 700.3 cm^−1^; ${\left[\alpha \right]}_{D}^{25}$ = −34.5 (0.38, MeOH).

*Methyl (R)-2-((S)-3-(dibenzylamino)-2-oxoazetidin-1-yl)-3-phenylpropanoate* (**3c*_R_***, Scheme 2). Yellow oil (9% yield); R_f_ (hexane/ethyl acetate = 6/1) = 0.13; ^1^H-NMR (400 MHz, CDCl_3_): *δ* 7.38–7.08 (m, 15H), 4.85 (dd, *J* = 11.3, 5.1 Hz, 1H), 4.25 (dd, *J* = 5.0, 2.4 Hz, 1H), 3.72 (s, 3H), 3.40, 3.24 (AB, *J* = 14.0 Hz, 4H), 3.34, 3.11 (AMX, *J* = 5.5, 5.0, 2.4 Hz, 1H), 3.31, 2.95 (AMX, *J* = 14.6, 5.1, 11.3 Hz 2H) ppm; ^13^C-NMR (100 MHz, CDCl_3_): δ 170.7, 169.5, 138.5, 136.2, 129.0 (2 signals), 128.9, 128.5, 127.4, 127.3, 68.7, 54.4, 53.8, 52.7, 42.7, 36.0 ppm; HRMS (ESI^−^): Calc mass for C_27_H_29_O_3_N_2_ [M − H]^−^: 429.2173; measured: 429.2172; IR (ATR): 3332.8, 3027.8, 2952.5, 1736.2, 1453.9, 1259.3, 1221.4, 1096.2, 1027.9, 735.4, 697.0 cm^−1^.

#### 3.2.5. Synthesis of Chlorides **6a** and **6b** (Scheme 2)

Compounds **6a** and **6b** were synthesized according to the procedures described for the preparation of **5** using SO_2_Cl_2_ or CH_3_SO_2_Cl to obtain a crude mixture, which was purified with flash column chromatography. However, a mixture of both compounds **6a** and **6b** in equimolar ratio was obtained, which was further purified with preparative HPLC. A separation was possible, but the compounds were transformed back upon treatment to give mixture of **6a**:**6b** in a ratio 1:1 again. The reported data are given for a mixture of both compounds. Colorless liquid (60% yield); R_f_ (hexane/diethyl ether = 3/1) = 0.36; ^1^H-NMR (400 MHz, acetone-*d*_6_): *δ* 7.84 (d, *J* = 7.9 Hz, 1H), 7.65 (d, *J* = 8.0 Hz, 1H), 4.82 (ddd, *J* = 8.8, 8.0, 5.1 Hz, 1H), 4.72 (ddd, *J* = 8.1, 7.9, 5.6 Hz, 1H), 4.47 (dd, *J* = 6.9, 6.4 Hz, 1H), 4.00, 3.88 (ABX, *J* = 11.3, 6.8, 6.7 Hz, 2H), 3.75, 3.53 (AX, *J* = 13.9 Hz, 4H), 3.71, 3.58 (AB, *J* = 13.7 Hz, 4H), 3.50 (dd, *J* = 6.7, 6.8 Hz, 1H), 3.28, 3.08 (AMX, *J* = 14.0, 8.8, 5.1 Hz, 2H), 3.18, 3.07 (ABX, *J* = 13.9, 5.6, 8.1 Hz, 2H), 3.12, 2.83 (AMX, *J* = 13.5, 6.9, 6.4 Hz, 2H,) ppm; ^13^C-NMR (100 MHz, acetone-*d*_6_): *δ* 172.6, 172.2, 170.0, 168.6, 140.1, 139.8, 138.0, 137.8, 130.3, 129.9, 129.7, 129.4, 129.3, 129.2, 129.1, 128.0, 127.7, 64.6, 58.9, 58.3, 56.6, 55.1, 55.0, 54.4, 52.3, 52.5, 42.3, 38.2, 38.0 ppm; MS (ESI^+^): *m*/*z* 429 [M + H]^+^; HRMS (ESI^+^): Calc mass for C_27_H_29_ClNO_3_ [M + H]^+^: 465.1939; measured: 465.1928; IR (ATR): 3028.6, 1737.1, 1603.2, 1494.4, 1453.1, 1377.9, 1256.4, 1221.8, 1174.0, 1128.7, 1026.1, 915.4, 745.9, 698.7 cm^−1^

#### 3.2.6. Synthesis of Tyrosine Derivatives **1d**–**1f** (Scheme 3)

*Methyl (R)-3-(4-(benzyloxy)phenyl)-2-((tert-butoxycarbonyl)amino)propanoate* (**S8a**) [29]. Benzyl protected compound **S8a** was synthesized according to the modified literature procedure [29] from methyl (*tert*-butoxycarbonyl)-d-tyrosinate (**S7**) (8.80 g, 29.8 mmol, 1 eq) which was dissolved in acetone (160 mL). Potassium carbonate (4.12 g, 29.8 mmol, 1 eq) was added and the mixture obtained was cooled to 0 °C followed by dropwise addition of benzyl bromide (3.9 mL, 32.9 mmol, 1.1 eq) and the reaction mixture was stirred for 24 h at room temperature. Ethyl acetate (200 mL) was then added and the solution was washed with water (100 mL). Water layer was then extracted with ethyl acetate (2 × 50 mL) and the combined organic fractions were washed with brine, dried over anhydrous sodium sulfate, filtered and evaporated. A crude mixture was purified with flash column chromatography using hexane/ethyl acetate = 3:1 as eluent. White amorphous solid (10.77 g, 27.9 mmol, 94% yield); mp = 83–86 °C; R_f_ (hexane:ethyl acetate = 3:1) = 0.38; ^1^H-NMR (400 MHz, CDCl_3_): *δ* 7.45–7.28 (m, 5H), 7.03 (d, *J* = 8.6 Hz, 2H), 6.90 (d, *J* = 8.6 Hz, 2H), 5.03 (s, 2H), 4.98 (d, *J* = 8.0 Hz, 1H), 4.54 (ddd, *J* = 8.0, 6.1, 5.7 Hz, 1H), 3.70 (s, 3H), 3.05, 2.99 (ABX, *J =* 14.1, 6.1, 5.7 Hz, 2H), 1.42 (s, 9H) ppm; ^13^C-NMR (100 MHz, CDCl_3_): *δ* 172.5, 157.9, 155.2, 137.1, 130.4, 128.6, 128.3, 128.0, 127.5, 114.9, 79.9, 70.0, 54.6, 52.2, 37.4, 28.4 ppm; MS (ESI^+^): 408 [M + Na]^+^; IR (ATR): 3367.2, 2977.4, 1742.6, 1710.4, 1509.8, 1365.4, 1241.0, 1161.1, 1016.9, 732.9 cm^−1^; ${\left[\alpha \right]}_{D}^{25}$ = −4.3 (0.66, MeOH).

*Methyl (R)-3-(4-acetoxyphenyl)-2-((tert-butoxycarbonyl)amino)propanoate* (**S8b**) **[30]**. Acetyl protected compound **S8b** was synthesized from methyl (*tert*-butoxycarbonyl)-d-tyrosinate (**S7**) (8.02 g, 27.2 mmol, 1 eq) which was dissolved in pyridine (150 mL) and the mixture obtained was cooled to 0 °C followed by dropwise addition of acetanhydride (12.7 mL, 136 mmol, 5 eq) and the reaction mixture was stirred for 24 h at room temperature. Ethyl acetate (200 mL) was then added and the solution was washed with 1 M HCl (2 × 50 mL), saturated NaHCO_3_ (2 × 50 mL) and brine (50 mL). Organic fraction was dried over anhydrous sodium sulfate, filtered and evaporated. A crude mixture was purified by crystallization from diethyl ether. White amorphous solid (8.65 g, 25.5 mmol, 94% yield); mp = 54–56 °C; ^1^H-NMR (400 MHz, DMSO-*d*_6_): *δ* 7.26 (d, *J* = 8.4 Hz, 2H), 7.03 (d, *J* = 8.4 Hz, 2H), 4.17 (ddd, *J* = 10.2, 8.1, 5.0 Hz, 1H), 3.61 (s, 3H), 2.99, 2.85 (ABX, *J* = 13.8, 10.4, 5.0 Hz, 2H), 2.25 (s, 3H), 1.32 (s, 9H) ppm; ^13^C-NMR (100 MHz, CDCl_3_): *δ* 173.0, 169.7, 155.9, 149.6, 130.5, 122.0, 78.8, 55.6, 52.3, 36.2, 28.6, 21.3 ppm; MS (ESI^+^): 359.9 [M + Na]^+^; IR (ATR): 3375.4, 2980.2, 1748.4, 1684.6, 1502.1, 1368.3, 1216.5, 1163.9, 1022.4 cm^−1^; ${\left[\alpha \right]}_{D}^{25}$ = +5.3 (0.56, 8.5 mg CH_3_CN).

*Methyl (R)-2-(4-(benzyloxy)phenyl)-2-((tert-butoxycarbonyl)amino)acetate* (**S8c**) [31]. Benzyl protected compound **S8c** was synthesized from methyl (*R*)-2-(4-(benzyloxy)phenyl)-2-((*tert*-butoxycarbonyl) amino)acetate (**S7c**) by the literature described procedure [31]. Yelow oil (8.735 g, 23.5 mmol, 98% yield); R_f_ (Hexane/ethyl acetate = 1/1) = 0.64; ^1^H-NMR (400 MHz, CDCl_3_) δ 1.43 (s, 9H), 3.71 (s, 3H), 5.05 (s, 2H), 5.25 (d, *J* = 7,1 Hz, 1H), 6.95 (d, *J* = 8.7 Hz, 2H), 7.27 (d, *J* = 8.7 Hz, 2H), 7.33−7.43 (m, 6H) ppm.

*(R)-3-(4-(Benzyloxy)phenyl)-1-methoxy-1-oxopropan-2-aminium trifluoroacetate* (**S9a**) [31]. Amine **S9a** was synthesized according to the modified literature procedure for *S* enantiomer [32] from **S8a** (10.6 g, 27.5 mmol, 1 eq), which was dissolved in CH_2_Cl_2_ (90 mL). Trifluoroacetic acid (8.4 mL, 110 mmol, 4 eq) was added and after 18 h of stirring overnight, the solution was evaporated and crystalized from diethyl ether. White amorphous solid (9.58 g, 24.0 mmol, 87% yield); mp = 117–119 °C; ^1^H-NMR (400 MHz, DMSO-*d*_6_): *δ* 8.45 (s, 3H), 7.47–7.30 (m, 5H), 7.14 (d, *J* = 8.6 Hz, 2H), 6.99 (d, *J* = 8.6 Hz, 2H), 5.09 (s, 2H), 4.54 (t, *J* = 6.5 Hz, 1H), 3.69 (s, 3H), 3.01, 3.06 (ABX, *J =* 14.2, 6.5 Hz, 2H) ppm; MS (ESI^+^): 286 [M + H]^+^; IR (ATR): 3034.0, 1742.1, 1662.6, 1610.9, 1513.1, 1449.0, 1243.1, 1179.3, 1135.1, 836.8, 721.2 cm^−1^; ${\left[\alpha \right]}_{D}^{25}$ = −15.4 (0.45, MeOH).

*(R)-3-(4-Acetoxyphenyl)-1-methoxy-1-oxopropan-2-aminium trifluoroacetate* (**S9b**) [30]. Amine **S9b** was synthesized according to the modified literature procedure for *S* enantiomer [30] from **S8b** (10.6 g, 27.5 mmol, 1 eq), which was dissolved in CH_2_Cl_2_ (90 mL). Trifluoroacetic acid (8.4 mL, 110 mmol, 4 eq) was added and after 18 h of stirring overnight, the solution was evaporated and used in further reactions without purification.

*(R)-1-(4-(Benzyloxy)phenyl)-2-methoxy-2-oxoethan-1-aminium chloride* (**S9c**) [33]. Amine **S9c** was synthesized according to the modified literature procedure [33] from **S8c**. White powder (6.869 g, 23.5 mmol, 93% yield); R_f_ (Hexane/ethyl acetate = 1/1) = 0.07; ^1^H-NMR (400 MHz, DMSO-*d*_6_): *δ* 3.38 (s, 3H), 3.70 (s, 3H), 5.14 (s, 2H), 5.20 (s, 1H), 7.08 (d, *J* = 8.8 Hz, 2H), 7.27−7.45 (m, 7H) ppm.

*Methyl (R)-3-(4-(benzyloxy)phenyl)-2-((S)-2-(dibenzylamino)-3-hydroxypropanamido) propanoate* (**1d**, Scheme 3). Dipeptide methyl dibenzyl-l-seryl-l-phenylalaninate (**1d**) was synthesized in a coupling procedure similar to the synthesis of **S3** starting from dibenzyl-l-serine (**S6**) (7.02 g, 24.6 mmol, 1.05 eq) and **S9** (9.33 g, 23.4 mmol, 1.00 eq). Yellow liquid (7.53 g, 77% yield); R_f_ (Hexane:ethyl acetate = 1:2) = 0.45; ^1^H-NMR (400 MHz, CDCl_3_): *δ* 7.74 (d, *J* = 8.9 Hz, 1H), 7.49–7.42 (m, 2H), 7.41–7.34 (m, 2H), 7.34–7.20 (m, 7H), 7.19–7.11 (m, 4H), 6.99–6.90 (m, 4H), 5.08, 5.04 (AB, *J* = 11.8 Hz, 2H), 4.85 (ddd, *J =* 8.9, 5.3, 5.5 Hz, 1H), 4.11, 3.97 (ABX, *J* = 11.4, 7.5, 4.0 Hz, 2H), 3.84, 3.43 (AB, *J* = 13.3 Hz, 4H), 3.62 (s, 3H), 3.30 (dd, *J* = 7.5, 4.0 Hz, 1H), 3.12, 2.98 (ABX, *J* = 13.7, 5.6, 5.2 Hz, 2H) ppm; ^13^C-NMR (100 MHz, CDCl_3_): *δ* 174.1, 171.8, 158.3, 138.5, 136.9, 130.5, 128.9, 128.8, 128.6, 128.2, 127.8, 127.6 (2 peaks), 115.2, 70.1, 61.7, 57.4, 54.7,52.6, 52.4, 36.8 ppm; MS (ESI^+^): *m*/*z* 575 [M + Na]^+^; MS (ESI^−^): *m*/*z* 551 [M − H]^+^; HRMS (ESI^−^): Calc mass for C_34_H_35_N_2_O_5_ [M − H]^−^: 551.2546; measured: 551.2542; IR (ATR): 3367.6, 3029.7, 1741.7, 1662.7, 1509.0, 1241.3, 1176.2, 1026.8, 909.0, 729.8, 696.3 cm^−1^; ${\left[\alpha \right]}_{D}^{25}$ = +17.4 (c 1.0, MeOH).

*Methyl (S)-2-(4-(benzyloxy)phenyl)-2-(2-(dibenzylamino)-3-hydroxypropanamido)acetate* (**1e**, Scheme 3). Dipeptide methyl (*S*)-2-(4-(benzyloxy)phenyl)-2-(2-(dibenzylamino)-3-hydroxypropanamido) acetate (**1e**) was synthesized as a mixture of diastereoisomers in a coupling procedure similar to the synthesis of **S3** starting from dibenzyl-l-serine (**S6**) (10.0 g, 35.0 mmol, 1 eq) and **S9c** (12.96 g, 42.0 mmol, 1.20 eq). Orange oil (10.368 g, 19.3 mmol, 55% yield); R_f_ (Hexane/ethyl acetate = 1/1) = 0.31; ^1^H-NMR (400 MHz, CDCl_3_): *δ* 8.46 (d, *J* = 7.1 Hz, 0.5H), 8.37 (d, *J* = 7.4 Hz, 0.5H), 7.27−7.42 (m, 15H), 7.20 (d, *J* = 8.7 Hz, 1H), 7.13 (d, *J* = 8.7 Hz, 1H), 6.89 (dd, *J* = 13.2, 8.7 Hz, 2H), 5.44 (d, *J* = 2.2 Hz, 0.5H), 5.42 (d, *J* = 2.2 Hz, 0.5H), 5.05 (s, 1H), 5.02 (s, 1H), 4.14−4.17 (m, 0,5H), 4.04−4.08 (m, 0.5H), 3.93−4.03 (m, 2H), 3.84 (d, *J* = 13.6 Hz, 1H), 3.75 (s, 1.5H), 3.73 (s, 1.5H), 3.57 (d, *J* = 4.1 Hz, 1H), 3.53 (d, *J* = 4.5 Hz, 1H), 3.40 (dd, *J* = 7.5, 4.0 Hz, 0.5H), 3.34 (dd, *J* = 7.5, 3.7 Hz, 0.5H), 3.25−3.31 (m, 1H) ppm; ^13^C-NMR (100 MHz, MeOD): *δ* 173.6; 171.2; 158.9; 138.5; 136.7; 128.9; 128.7; 128.5; 128.4; 128.3; 128.1; 127.6; 127.5; 115.3; 70.1; 62.1; 57.8; 55.7; 54.8; 52.9 ppm; MS (ESI^+^): 561 [M + Na]^+^; 537.2 [M − H]^−^; HRMS (ESI^−^): Calc mass for C_33_H_33_N_2_O_5_ [M − H]^−^: 537.2395; measured: 537.2399; IR (ATR): 3480, 3363, 3032, 2951, 1728, 1666, 1608, 1505, 1453, 1435, 1383, 1302, 1247, 1173, 1138, 1079, 1012, 992, 905, 855, 806, 743, 700, 646, 609, 575, 524, 505 cm^−1^. 

*Methyl (R)-3-(4-acetoxyphenyl)-2-((S)-2-(dibenzylamino)-3-hydroxypropanamido)propanoate* (**1f**, Scheme 3). Dipeptide methyl dibenzyl-l-seryl-l-phenylalaninate (**1f**) was synthesized in a coupling procedure similar to the synthesis of **S3** starting from dibenzyl-l-serine (**S6**) (12.14 g, 24.6 mmol, 1.3 eq) and **S9b** (8.50 g, 32.7 mmol, 1.00 eq) [30]. White crystals (8.32 g, 52% yield); mp =116–117 °C; R_f_ (Hexane: ethyl acetate = 1:1) = 0.32; ^1^H-NMR (400 MHz, CDCl_3_): *δ* 7.76 (d, *J* = 8.8 Hz, 1H), 7.35–7.14 (m, 10H), 7.04–6.98 (m, 4H), 4.88 (ddd, *J =* 8.8, 5.6, 5.6 Hz, 1H), 4.14, 4.01 (ABX, *J* = 11.4, 7.5, 4.6 Hz, 2H), 3.88, 3.51. (AB, *J* = 13.4 Hz, 4H), 3.66 (s, 3H), 3.34 (dd, *J* = 7.4, 3.9 Hz, 1H), 3.13, 3.09 (ABX, *J* = 14.0, 5.8, 5.4 Hz, 2H), 2.35 (s, 3H) ppm; ^13^C-NMR (100 MHz, CDCl_3_): *δ* 174.0, 171.6, 169.5, 150.0, 138.5, 133.6, 130.4, 128.9, 128.7, 127.6, 122.0, 61.8, 57.5, 54.7, 52.6, 52.5, 37.3, 21.3 ppm; HRMS (ESI^+^): Calc mass for C_29_H_33_N_2_O_6_ 505.2333 [M + H] ^+^; measured: 505.2335; IR (ATR): 3460.3, 3328.0, 1758.3, 1738.2, 1669.4, 1669.4, 1503.7, 1215.6, 1194.9, 914.7, 755.4, 701.8, 585.5 cm^−1^; ${\left[\alpha \right]}_{D}^{25}$ = −99.0 (c 0.546, MeOH).

*Methyl (R)-3-(4-(benzyloxy)phenyl)-2-((S)-3-(dibenzylamino)-2-oxoazetidin-1-yl)propanoate* (**3d**, Scheme 3). β-Lactam **3d** was synthesized from dipeptide **1d** (7.37 g, 13.3 mmol) according to the procedure described for the preparation of **3c** using PPh_3_ and DIAD in THF to obtain a crude mixture, which was purified with flash column chromatography on silica using petroleum ether:acetone = 10:3 as eluent. White crystals (7.06 g, 99% yield); R_f_ (petroleum ether:acetone = 10:3) = 0.22; mp = 92–93 °C, ^1^H-NMR (400 MHz, CDCl_3_) δ 7.41–7.17 (m, 15H), 7.13 (d, *J* = 8.7 Hz, 2H), 6.82 (d, *J* = 8.7 Hz, 2H), 4.84 (dd, *J* = 11.5, 5.1 Hz, 1H), 4.72, 4.69 (AB, *J* = 11.7 Hz, 2H), 4.25 (dd, *J* = 5.1, 2.3 Hz, 1H), 3.72 (s, 3H, 3.38, 3.22 (AB, *J* = 13.7 Hz, 4H), 3.36, 3.10 (AMX, *J* = 5.6, 5.1, 2.3 Hz, 2H), 3.26, 2.89 (AMX, *J* = 14.7, 5.1, 11.5 Hz, 2H) ppm; ^13^C-NMR (100 MHz, CDCl_3_): δ 170.7, 169.4, 158.0, 138.4, 136.9, 129.8, 128.9, 128.6, 128.3, 128.2, 128.0, 127.5, 127.2, 115.1, 69.7, 68.7, 54.2, 53.5, 52.5, 42.5, 35.1 ppm; HRMS (ESI^+^): Calc mass for C_34_H_35_N_2_O_4_: 535.2597; measured: 535.2596 [M + H]^+^; IR (ATR): 3443.2, 1743.8, 1665.6, 1496.5, 1195.6, 746.4, 699.5 cm^−1^; ${\left[\alpha \right]}_{D}^{25}$ = +59.5 (c 0.347, MeOH).

*Methyl (R)-2-(4-(benzyloxy)phenyl)-2-(3-(dibenzylamino)-2-oxoazetidin-1-yl)acetate* (**3e**, Scheme 3). β-Lactam **3e** was synthesized from dipeptide **1e** (9.12 g, 34.8 mmol) according to the procedure described for the preparation of **3c** using PPh_3_ and DIAD in THF to obtain a crude mixture, which was purified with flash column chromatography on silica using hexane/ethyl acetate = 2/1 as eluent to obtain diastereoisomeric mixture of products. Yellow gel (7.30 g, 14.0 mmol, 32% yield), R_f_ (Hexane/ethyl acetate = 2/1) = 0.26; ^1^H-NMR (400 MHz, MeOD) *δ* 7.17–7.43 (m, 17H), 6.99–7.02 (m, 2H), 5.54 (s, 0.4H), 5.53 (s, 0.6H), 4.31 (dd, *J* = 5.0, 2.4 Hz, 0.4H), 4.19 (dd, *J* = 5.1, 2.4 Hz, 0.6H), 3.77–3.80 (m, 2H), 3.71–3.72 (m, 3H), 3.63–3.69 (m, 2H), 3.55 (dd, *J* = 5.7, 2.3 Hz, 0.6H), 3.51 (d, *J* = 13.5 Hz, 2H), 3.46 (t, *J* =5.2 Hz, 0.4H), 3.02 (dd, *J* = 5.9, 2.4 Hz, 0.4H), 2.92 (t, *J* = 5.4 Hz, 0.6H) ppm; ^13^C-NMR (100 MHz, MeOD): *δ* 171.5; 171.1; 160.6; 139.6; 139.4; 138.4; 130.8; 130.2; 130.2; 129.6; 129.4; 129.0; 128.6; 128.4; 128.4; 127.0; 126.6; 116.5; 116.5; 71.1; 69.2; 61.6; 58.5; 58.4; 55.7; 55.5; 53.2; 44.19; 43.8 ppm; MS (ESI^+^): 542.7 [M + (Na)]^+^; IR (ATR): 3245.3, 3033.1, 2983.6, 1734.7, 1688.2, 1610.7, 1513.3, 1454.1, 1378.2, 1255.5, 1177.6, 1109.4, 1052.1, 1023.3, 925.3, 852.2, 793.7, 743.8, 699.4, 634.2, 597.1 cm^−1^; HRMS (ESI^+^): Calc mass for C_33_H_33_N_2_O_4_ [M + H]^+^: 521.2435; measured: 521.2432.

*Methyl (R)-3-(4-acetoxyphenyl)-2-((S)-3-(dibenzylamino)-2-oxoazetidin-1-yl)propanoate* (**3f**, Scheme 3). β-Lactam **3f** was synthesized from dipeptide **1f** (91 mg, 180 μmol) according to the procedure described for the preparation of **3c** using PPh_3_ and DIAD in THF to obtain a crude mixture, which was purified with flash column chromatography on silica using CH_2_Cl_2_:acetone = 50:1 as eluent. Viscous oil (139 mg, 90% yield); R_f_ (CH_2_Cl_2_:acetone = 50:1) = 0.17; ^1^H-NMR (400 MHz, CDCl_3_): *δ* 7.34–7.18 (m, 12H), 6.99 (d, *J* = 8.5 Hz, 2H), 4.77 (dd, *J* = 10.7, 5.4 Hz, 1H), 4.25 (dd, *J* = 5.1, 2.4 Hz, 1H), 3.70 (s, 3H), 3.48, 3.34 (AB, *J* = 13.4 Hz, 4H), 3.32, 3.10 (AMX, *J* = 5.5, 5.1, 2.5 Hz, 2H), 3.27, 2.98 (AMX, *J* = 14.7, 10.8, 5.5 Hz, 2H) ppm; ^13^C-NMR (100 MHz, CDCl_3_): δ 170.4, 169.4, 169.3 (2 signals overlapping), 149.8, 138.3, 133.7, 129.8, 129.0, 128.4, 127.2, 121.9, 68.7, 54.5, 54.0, 52.6, 42.9, 35.2 ppm; HRMS (ESI^+^): Calc mass for C_29_H_31_O_5_N_2_ [M + H]^+^: 487.2227, measured: 487.2221; IR (ATR): 2952.4, 1735.1, 1508.6, 1369.8, 1369.8, 1193.5, 1104.6, 1018.3, 748.1, 699.0 cm^−1^; ${\left[\alpha \right]}_{D}^{25}$ = +61.2 (0.623, MeOH).

*Methyl (R)-2-((S)-3-(dibenzylamino)-2-oxoazetidin-1-yl)-3-(4-hydroxyphenyl)propanoate* (**8**, Scheme 3). β-Lactam **8** was synthesized from acetate **3f** (4.30 g, 8.84 mmol) in MeOH (88 mL) to which a 1 M aqueous solution of LiOH (10.60 mL, 10.6 mmol, 1.2 eq) was added dropwise at 0 °C during 3 h. The solution was neutralized with acetic acid and evaporated. Viscous oil (3.53 g, 90% yield); R_f_ (CH_2_Cl_2_:acetone = 30:1) = 0.33; ^1^H-NMR (400 MHz, CDCl_3_): *δ* 7.31–7.24 (m, 8H, Ar-H), 7.23–7.17 (m, 2H, Ar-H), 6.99 (d, *J* = 8.41 Hz, 2H), 6.66 (d, *J* = 8.6 Hz, 2H), 4.78 (dd, *J* = 11.1, 5.3 Hz, 1H), 4.26 (dd, *J* = 5.0, 2.4 Hz, 1H), 3.69 (s, 3H), 3.45, 3.32 (AB, *J* = 13.4 Hz, 4H), 3.36, 3.16 (AMX, *J* = 5.5, 5.0, 2.4 Hz, 2H), 3.21, 2.87 (AMX, *J* = 14.6, 11.1, 5.3 Hz, 2H) ppm; ^13^C-NMR (100 MHz, CDCl_3_): *δ* 170.6, 170.2, 155.6, 138.2, 129.7, 128.8, 128.3, 127.2, 127.0, 115.9, 68.2, 54.3 (2 signals overlapping), 52.6, 43.0, 35.0 ppm; HRMS (ESI^+^): Calc mass for C_27_H_29_O_4_N_2_: 445.2122, measured: 445.2113 [M + H]^+^; IR (ATR): 3308.9, 3027.4, 2953.18, 1724.3, 1516.6, 1221.8, 1173.9, 731.7, 697.7 cm^−1^; ${\left[\alpha \right]}_{D}^{25\;}$ = +56.2 (c 0.623, MeOH).

#### 3.2.7. Deprotection of Dibenzylamine from **3c**–**3f** and **8** for the Preparation of **7c**–**7e** (Scheme 3)

*Methyl (S)-2-((S)-3-amino-2-oxoazetidin-1-yl)-3-phenylpropanoate* (**7c**, Scheme 3). Dibenzylamine **3c** (450 mg, 1.05 mmol) was dissolved in a mixture of ethyl acetate and ethanol (95:5) and the solution was flushed with argon. Palladium on activated carbon (5% Pd, 90 mg) was added and the suspension was slowly flushed with hydrogen for 30 min. The mixture was stirred under hydrogen for 24 h, palladium was then removed by filtration through a pad of Celite and the solution obtained evaporated. The crude product was purified with flash column chromatography on silica using CH_2_Cl_2_:MeOH = 20:1 as eluent. White crystals (156 mg, 0.63 mmol, 60% yield); mp = 274–276 °C; R_f_ (CH_2_Cl_2_:MeOH = 20:1) = 0.17; ^1^H-NMR (400 MHz, CDCl_3_): *δ* 7.34–7.15 (m, 5H), 4.85 (dd, *J* = 10.0, 5.4 Hz, 1H), 3.98 (dd, *J* = 5.1, 2.4 Hz, 1H), 3.74 (s, 3H), 3.11, 3.15 (AMX, *J* = 5.6, 5.1, 2.4 Hz, 2H), 3.24, 3.00 (AMX, *J* = 14.4, 10.0, 5.4 Hz 2H), 1.86 (bs, 2H) ppm; ^13^C-NMR (100 MHz, CDCl_3_): *δ* 171.2, 170.5, 136.2, 128.8, 128.7, 127.3, 59.5, 54.8, 52.6, 49.4, 35.7 ppm; MS (ESI^+^): *m*/*z* 271.2 [M + Na]^+^; HRMS (ESI^+^): Calc mass for C_13_H_17_N_2_O_3_: 249,1234; measured: 249.1232 [M + H]^+^; IR (ATR): 3325.0, 2954.3, 1731.2, 1631.2, 1554.1, 1380.2, 1340.3, 1222.7, 1174.7, 744.3, 698.7 cm^−1^; ${\left[\alpha \right]}_{D}^{25}$ = −41.8 (1.874, MeOH).

*Methyl (R)-2-((S)-3-amino-2-oxoazetidin-1-yl)-3-(4-hydroxyphenyl)propanoate* (**7d**, Scheme 3). Compound **7d** was synthesized from **3d** (4.30 g, 6.4 mmol) or **8** (2.00 g, 3.60 mmol) according to the procedure described for the preparation of **7c** and purified with flash column chromatography on silica using CH_2_Cl_2_:MeOH = 9:1 as eluent. Tetrahedral white crystals (from **3d**: 1.37 g, 5.2 mmol, 80% yield, from **8**: 808 mg, 3.1 mmol, 85% yield); mp = 94–95 °C; R_f_ (CH_2_Cl_2_:MeOH = 9:1) = 0.21; ^1^H-NMR (400 MHz, CDCl_3_): *δ* 7.07 (d, *J* = 8.5 Hz, 2H), 6.72 (d, *J* = 8.5 Hz, 2H), 4.74 (dd, *J* = 11.1, 4.9 Hz, 1H), 4.09 (dd, *J* = 5.1, 2.3 Hz, 1H), 3.85 (bs, 2H), 3.76 (s, 3H), 3.72, 3.01 (AMX, *J* = 5.8, 5.1, 2.3 Hz, 2H), 3.23, 2.89 (AMX, *J* = 14.5, 11.1, 4.8 Hz 2H) ppm; ^13^C-NMR (100 MHz, CDCl_3_): *δ* 171.4, 170.5, 156.1, 130.1, 126.9, 115.9, 59.2, 54.7, 52.7, 49.2, 35.3 ppm; HRMS (ESI^+^): Calc mass for C_13_H_17_N_2_O_4_ [M + H]^+^: 265.1188; measured: 265.1184; IR (ATR): 3260.4, 1731.1, 1649.4, 1514.8, 1229.5, 1019.9, 825.1 cm^−1^; ${\left[\alpha \right]}_{D}^{25}$ = +52.6 (0.61, MeOH).

*Methyl (R)-2-(3-amino-2-oxoazetidin-1-yl)-2-(4-hydroxyphenyl)acetate* (**7e**, Scheme 3). Compound **7e** was synthesized from **1e** (3.85 g, 7.4 mmol) according to the procedure described for the preparation of **7c** and purified with flash column chromatography on silica using CH_2_Cl_2_:MeOH = 9:1 as eluent. Yellow gel (1.444 g, 5.77 mmol, 78% yield); R_f_ (DKM/MeOH= 9/1) = 0.29; ^1^H-NMR (400 MHz, MeOD): *δ* 7.13–7.17 (m, 2H), 6.81–6.84 (m, 2H), 5.48 (s, 1H), 4.18 (dd, *J* = 5.3, 2.5 Hz, 0.5H), 4.06 (dd, *J* = 4.9, 2.5 Hz, 0.5H), 3.82 (t, *J* = 5.3 Hz, 0.5H), 3.75 (s, 3H), 3.37–3.39 (m, 1H), 2.85–2.88 (m, 0.5H) ppm; ^13^C-NMR (100 MHz, MeOD): *δ* 172.0; 171.7; 159.3; 130.8; 130.6; 125.2; 116.8; 59.6; 58.5; 53.1; 50.3 ppm; MS (ESI^+^): 251.1 [M + H]^+^; HRMS (ESI^+^): Calc mass for C_12_H_14_O_4_N_2_: 251.1032, measured: 251.1038 [M + H]^+^; IR (ATR): 2959.1, 1732.4, 1648.3, 1611.9, 1515.5, 1451.6, 1384.3, 1236.7, 1175.2, 1106.2, 1011.1, 833.4, 757.5, 528.9 cm^−1^; HRMS (ESI^−^): Calc mass for C_12_H_13_N_2_O_4_ [M − H]^−^: 249.0881; measured: 249.0884.

## 4. Conclusions

There are only a limited number of methodologies used for the preparation of C4-β-lactam–containing dipeptides without a substituent on C4. A study was conducted here of the different reaction conditions on serine dipeptides that were *N*-protected with a phthalimide, trityl, or dibenzyl group. This demonstrated that the protective group and the choice of correct reaction conditions are very important for the successful formation of the β-lactam ring, and for the extent of side-product formation. Across all of the reaction conditions that were investigated, the use of the Mitsunobu reaction on dibenzyl-protected dipeptides gave the best results. Importantly, the dibenzyl group was easily removed from the β-lactam phenylalanine derivatives, and products with a free amino group were obtained. The present approach adds to the utility of the *N*-dibenzyl protective group for the synthesis of β-lactam phenylalanine-containing dipeptides.
